# Mechanisms of innate immune cells in Metabolic dysfunction-associated steatotic liver disease

**DOI:** 10.3389/fimmu.2025.1599748

**Published:** 2025-09-25

**Authors:** Yao Yao, Qiong Wu, Bin Fan, Xin Peng, Mingwei Sheng, Fengmei Wang

**Affiliations:** ^1^ Department of Gastroenterology and Hepatology, Tianjin First Central Hospital, Nankai University, Tianjin, China; ^2^ Department of Urology, Tianjin First Central Hospital, Nankai University, Tianjin, China; ^3^ Tianjin Key Laboratory of Radiation Medicine and Molecular Nuclear Medicine, Institute of Radiation Medicine, State Key Laboratory of Advanced Medical Materials and Devices, Tianjin Institutes of Health Science, Chinese Academy of Medical Sciences & Peking Union Medical College, Tianjin, China; ^4^ Department of Anesthesiology, Tianjin First Central Hospital, Nankai University, Tianjin, China

**Keywords:** MASLD, MASH, HCC, innate immune cells, ATMS

## Abstract

With the continuous increase of the incidence of Metabolic dysfunction-associated steatotic liver disease(MASLD), the proportion of MASLD-driven hepatocellular carcinoma(HCC) is gradually increasing, which will become a heavy burden on global public health. This article summarizes the existing literature and discusses the role of various innate immune cells in the occurrence and development of MASLD, such as cell-cell crosstalk, and uses bibliometric analysis to find current research hotspots and emerging topics, in order to provide valuable reference for scholars studying the direction of MASLD-driven HCC immunity. It also provides a solid foundation for more researchers to join the research direction. And try to inspire researchers in future research to seek breakthroughs in this regard.

## 1 Introduction

MASLD, formerly known as Non-Alcoholic Fatty Liver Disease (NAFLD), is defined as excess triglyceride storage in the liver in the presence of at least one cardiometabolic risk factor, including isolated metabolic dysfunction-associated steatotic liver(MASL), metabolic dysfunction-associated steatotic hepatitis (MASH), and fibrosis and cirrhosis (1). According to statistics, about 30% of the global population suffers from MASLD (2). Moreover, the incidence of MASH may increase by 56% in the next 10 years (3), and the overall burden of end-stage liver disease caused by MASLD may increase by 2 to 3 times in the next 20 years (4). Currently, approximately 2% of MASH converts to MASH-driven HCC each year (5). It is noteworthy that the progression process can also occur in the absence of cirrhosis (6). With the promotion and implementation of hepatitis B blocking therapy and the gradual improvement of clinical cure rate of hepatitis B, the proportion of MASLD-driven HCC is gradually increasing, which will be a heavy burden on global public health. Based on above, how to reduce the incidence of MASH and how to alleviate or reverse MASH-HCC are challenging point for scientific researchers.

It is well known that chronic inflammation is an important trigger for hepatocyte transformation and carcinogenesis, and that extensive liver inflammation is regulated by changes in innate immune activation. The inflammatory cascade in MASLD is caused by innate immunity, which involves myeloid innate immune cells such as macrophages (resident and recruited), monocytes, dendritic cells (DCs), neutrophils, eosinophils, basophils, and innate lymphoid cells(ILCs), and adaptive immunity, which involves B and T lymphocytes (7–9). Suppressing the excessive inflammatory response by regulating innate immune cells seems to be a feasible way forward.

**Figure 1:**
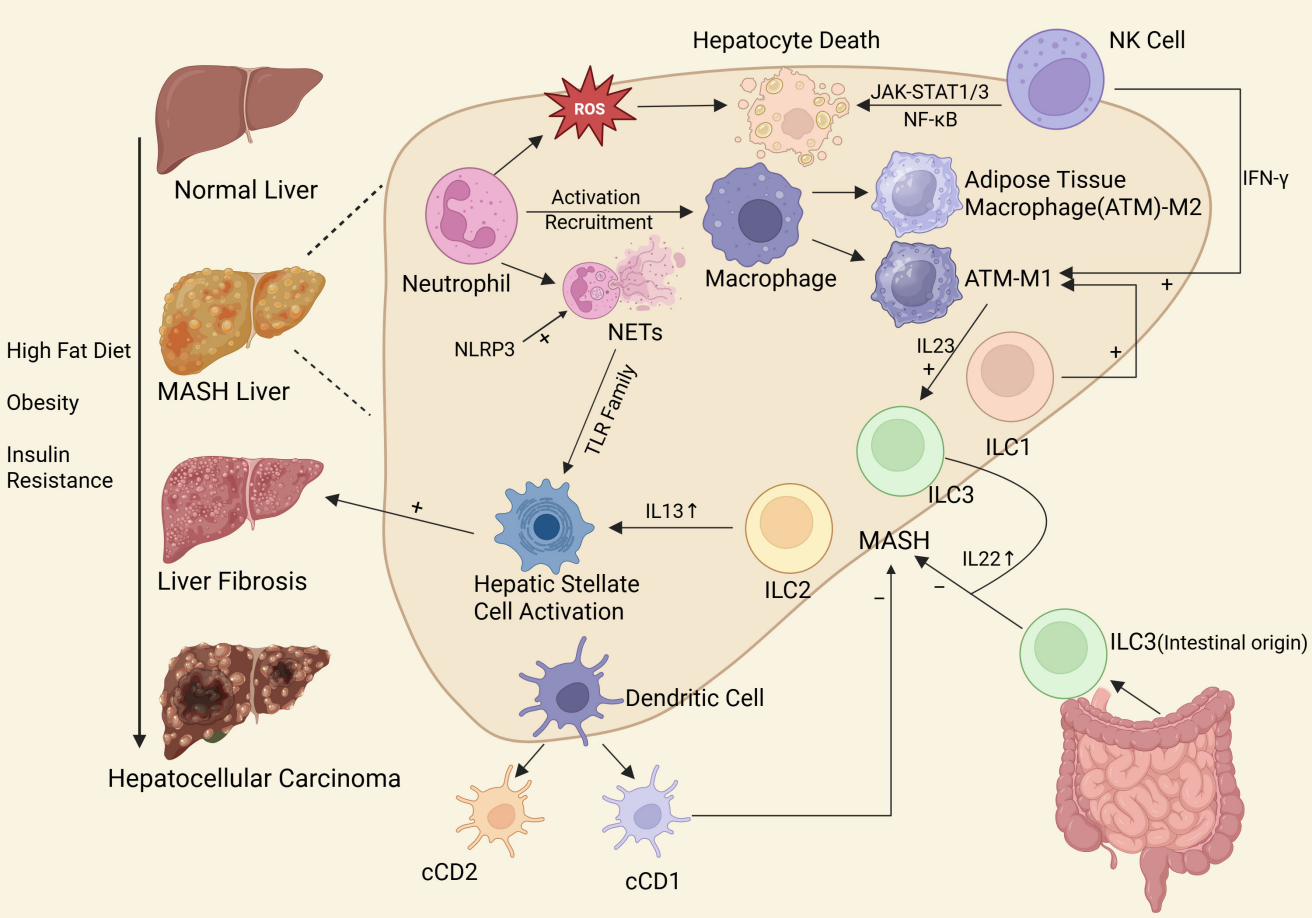
The role of innate immune cells in MASLD.

With the increasing incidence of MASLD and the proportion of MASLD-driven HCC worldwide, scholars from all over the world have carried out in-depth studies on the mechanisms of inflammation and cancer transformation of MASLD, especially the role of innate immune cells. Based on this, we have summarized the role of various innate immune cells in the occurrence and development of MASLD (**Figure 1**), searched the research on innate immune cells in MASLD published from 2000 to 2025, used bibliometric analysis to explore the current research hotspots and emerging topics, in order to provide a solid foundation for more researchers to join the research in this direction. And we aim to inspire researchers in future studies to seek breakthroughs in this area.

## 2 The role of various types of innate immune cells in the progression of MASLD

### 2.1 Macrophages

Macrophages were first identified by Elie Metchnikoff in 1884 (10). In 1876, Wilhelm von Kupffer (11) first discovered that there were a large number of tissue macrophages in the healthy liver, and these cells were named Kupffer cells (KCs). Hepatic macrophages are composed of KCs and recruited monocyte-derived macrophages. Under steady-state conditions, KCs comprise more than 90% of all hepatic macrophages, while monocytes circulate in the hepatic sinusoids but do not participate in the adult KCs repertoire. In the case of KCs depletion, bone marrow derived monocytes are rapidly recruited, colonize the liver, and produce monocyte-derived KCs that are genetically and functionally nearly identical to embryonic macrophages and acquire the ability to self-sustain in the liver without the subsequent contribution of bone marrow monocytes. Together, they constitute the largest population of innate immune cells in the liver and play a central role in maintaining liver homeostasis (12, 13).

According to the state of the microenvironment, liver macrophages can be differentiated into two different types, namely the classically activated pro-inflammatory M1 and the alternatively activated anti-inflammatory M2. In the context of MASLD, macrophage polarization has been reviewed and elucidated, which can be summarized as follows (14): In the early stage of MASH, resident KCs mainly promote triglyceride storage and exhibit an anti-inflammatory M2 phenotype. Depletion of KCs is beneficial for improve hepatocyte steatosis and insulin resistance, followed by the conversion of monocyte-derived macrophages into KCs within the hepatic sinusoids. However, monocyte-differentiated KCs can reduce lipid accumulation but exacerbate liver inflammation. Since the role of KCs in MASLD spectrum disorders has been reviewed in detail, this article focuses on the role of Adipose tissue macrophages(ATMs) in MASLD spectrum disorders, to supplement the immune role of macrophages in liver inflammation.

MASH pathology is characterized by hepatic fat accumulation and inflammatory cell infiltration, and although MASH occurs in the context of altered metabolism, there is a strong immuno-inflammatory component in the expanding adipose tissue compartment (15). ATMs, which constitute the majority of immune cells in adipose tissue, regulate tissue homeostasis in the lean state and metabolic dysregulation in obesity (16). Meanwhile, ATMs accumulate in high caloric intake environments and undergo transcriptional and phenotypic changes, from regulating metabolic dysregulation to promoting tissue inflammation and aggravating metabolic disorders (17). Adipose tissue inflammation significantly contributes to MASH progression and may even be a prerequisite for MASH progression. Boesch et al. (18) found that ablation of ATMs or removal of visceral white adipose tissue reduced insulin resistance and liver inflammation in animal models. Interestingly, Boesch et al. (18) showed that ATMs dysfunction causes loss of vascular integrity in adipose tissue characterized by albumin extravasation into perivascular tissues, which promotes the development of adipose tissue and liver inflammation. This can lead to the progression of MASLD to a more severe phenotype. This further confirms that ATMs play different roles under different conditions. When MASH-ATMs exhibit proinflammatory and lipid-related phenotypes, they secrete small extracellular vesicles (sEVs) enriched in fibrogenic miRNAs miR-155 and miR-34a to promote liver fibrosis progression (19). Growth differentiation factor-15 (GDF-15) is an effective anti-obesity target, which prevents obesity by binding and activating its receptor in the central nervous system (20, 21). Using large human cohorts of obesity, type 2 diabetes, and MASLD states, as well as several experimental mouse models of obesity, insulin resistance, and MASLD-like conditions, L’homme et al. (22) found that GDF-15 production increased first in adipose tissue at the onset of obesity and T2D and subsequently in the liver during MASLD progression. Accumulation of ATMs in adipose tissue is responsible for elevated GDF-15 in obesity and T2D patients. Notably, inactivation of GDF-15 in ATMs reduced plasma GDF-15 concentrations in mice and aggravated the development of diet-induced obesity and hepatic complications, especially progression from steatosis to MASH. In addition, Martinez-Sanchez et al. (23), a Spanish clinical trial, collected adipose tissue and liver biopsies from 42 MASLD patients with different fibrosis stages and found that the degree of liver fibrosis in MASLD patients was significantly correlated with the abundance of proinflammatory ATMs, and the modulation of ATMs improved liver fibrosis.The above studies further indicate that ATMs play a non-negligible role in the progression of MASLD. Resident ATMs can regulate metabolic disorders in obesity, and when ATMs accumulate, they can accelerate metabolic disorders. However, whether ATMs dysfunction in MASLD is a cause or a consequence of MASLD is a common question that has not yet been concluded. It is discussed in the Journal of Hepatology and suggests a possible need for longitudinal sampling and analysis of adipose tissue in large MASLD cohorts, with further clarification of links to clinical outcome data (24). This is also the direction that future researchers need to explore deeply.

### 2.2 Neutrophil

Inflammatory infiltration is the pathological feature of MASLD. Neutrophils are the most abundant subtype of leukocyte in the blood circulation. As the first responders of inflammation, neutrophils are the first cellular defense line of the human innate immune system, and play an indispensable role in the evolution process from simple hepatic steatosis to NASH (25). Neutrophils are primarily recognized for their role as antigen-presenting cells, which directly activate KCs and endothelial cells and promote cellular adhesion, At the same time, they plays a role in antibacterial response and inflammatory injury by producing ROS, granzymes, cytokines and neutrophil extracellular traps (NETs) (26). However, abnormal overproduction of these mediators may lead to increased inflammation. Brinkman et al. (27) first reported in 2004 that neutrophils can expel their nuclear material and establish NETs, which can trap and kill bacteria. In the following two decades, its role in various non-infectious inflammation and cancer has been gradually discovered. In 2018, Dirk et al. (28) found that neutrophils were stimulated to form NETs in a MASH mouse model, and that inhibition of NETs significantly reduced macrophage infiltration, inflammatory cytokine production, and HCC development. However, the underlying mechanisms remain unclear.

Innate immunity involves signal transduction through pattern recognition receptors, such as Toll-like Receptors (TLRs), which play an indispensable role in the process of recognizing pathogen-associated molecular patterns or damage-associated molecular patterns (29). TLR3 is the most up-regulated TLR in the TLR family in hepatic stellate cells(HSCs) stimulated by NETs, and TLR3 signaling induces inflammation (increased COX-2 expression) and HSCs activation (increased α-SMA expression) and PGE2 production, which promotes liver inflammation and injury and accelerates MASH related liver fibrosis (30). In contrast, TLR3 knockout HSCs lost the ability to activate in response to NETs stimulation. It has been shown that ROS are a key factors in promoting HSC activation (31). During MASH progression, ROS are released from mitochondria into the cytoplasm, ROS accumulation promotes ferroptosis of hepatocytes, recruits neutrophils, and induces the production of NETs, while blockade of NETs can inhibit the progression of MASH (32). These findings support that recruitment of NETs promotes hepatic stellate cell activation and subsequent fibrosis development in MASH mice, that is, NETs play an important role in the pathogenesis of MASH fibrosis.

NOD-like receptor protein 3 (NLRP3) inflammasome is expressed in various cell types of the liver, including hepatocytes, HSCs, macrophages, and neutrophils, and has been identified as one of the triggers of liver inflammation in MASLD (33, 34). NLRP3 was induced and activated in the liver of MASH mice, and that NLRP3 inhibitors significantly reduced liver neutrophil infiltration and regulated MASH fibrosis progression (35). Meanwhile, NETs formation, liver injury, and fibrosis were attenuated after administration of NLRP3 inhibitors in mice fed a MASH + alcohol diet (36). How NLRP3 regulates neutrophil recruitment and NETs formation is still unclear. Breast regression protein 39 (BRP39, human homolog YKL-40) may be one of the key targets. BRP39 is a glycoprotein expressed by many cell types, including macrophages, neutrophils, fibroblasts, and epithelial cells. In NLRP3-induced MASH, BRP39 deficiency improved liver inflammation and fibrosis, and the activation of HSCs cells was significantly reduced, and the aggregation of Ly6C^+^ infiltrating macrophages and H3Cit^+^ neutrophils in the liver was significantly reduced (37). BRP39-deficient mice exhibit impaired chemotaxis and migration of circulating neutrophils and decreased expression of RNAs associated with immune activation, migration, and signaling responses in neutrophils. NLRP3 inhibitors have now been investigated in preclinical studies in metabolic and inflammatory-driven diseases (gout, cardiovascular disease, cancer, etc.), neurodegenerative diseases, and cryopyrin-associated periodic syndrome (CAPS). Although basic studies have shown the beneficial effects of NLRP3 inhibitors in MASLD spectrum disorders, there is still a gap in preclinical studies. Currently, there are no FDA-approved NLRP3-targeting inhibitors. MCC950, commonly used in basic research on MASLD spectrum disorders, is a direct NLRP3 inhibitor that shows promise but exhibits off-target effects. The clinical development of MCC950 in rheumatoid arthritis was discontinued due to hepatotoxicity. Hence the preclinical evaluation of MCC950 in the treatment of MASLD patients should be carefully evaluated (38).

NETs can also serve as a bridge between innate and adaptive immunity. While promoting innate immunity, NETs promote Treg differentiation through metabolic reprogramming of naive CD4 T cells, crosstalk with adaptive immunity, and further prevent the occurrence of MASH-HCC (39). Moreover, NETs can directly affect hepatocytes and aggravate MASH by promoting hepatocyte senescence, leading to liver fibrosis (40). In conclusion, the production of NETs by neutrophils is an important pathway to maintain the inflammatory state, exerting its effects after injury. However, excessive NETs constitute harmful factors, which can exacerbate liver injury and promote cancer progression. Probably due to the short time of NETs research (research began in the past 20 years), no NETs blockers have been used in preclinical studies.

### 2.3 ILCs

Innate lymphoid cells (ILCs) are a group of tissue-resident immune cells that lack T cell and B cell receptors. According to their developmental and functional trajectories, ILCs are divided into five subsets: natural killer (NK) cells, ILC1s, ILC2s, ILC3s, and lymphoid tissue-induced cells, but their grouping is still controversial (41).

#### 2.3.1 NK cell

During the progression of MASH, liver inflammation (provided by lipotoxicity, immune-inflammatory cells, and endotoxemia) affects the mechanistic process of NK cells development and functional maturation (42). In the late stage, persistent high lipids promote programmed necrosis of NK cells, which in turn promotes the progression of MASH to fibrosis (43). Patients with obesity, simple steatosis, and progressive liver fibrosis have a greatly increased risk of developing HCC due to reduced NK cell numbers and function (44).

Hepatic NK cells have a dual role in nonalcoholic fatty liver disease. On the one hand, they can amplify the inflammatory response in the MASH stage (45). On the other hand, they can inhibit the development of fibrosis by killing activated hepatic stellate cells (46), or they can improve fibrosis by regulating the fibrotic properties of other liver-resident immune cells (47), thus playing a key role in the liver. Activated hepatic NK cells in MASH exhibit robust secretion of proinflammatory cytokines, including interferon-γ(IFN-γ), interleukin-1β(IL-1β), interleukin-12(IL-12), CCL4, CCL5, and granulocyte-macrophage colony-stimulating factor(GM-CSF). The absence of NK cells or IFN-γ preventes the accumulation of proinflammatory macrophages in visceral adipose tissue and significantly improved insulin sensitivity, corroborating the fact that NK cells can amplify the inflammatory response during the MASH phase (48). Besides, these proinflammatory cytokines secreted by NK cells activate hepatic Janus Kinase-Signal Transducers and Activators of Transcription 1/3(JAK-STAT1/3) and nuclear factor kappa-B (NF-κB) signaling pathways to induce hepatocyte injury. It is worth mentioning that NK cell-derived IFN-γ plays a key role in maintaining the balance of the inflammatory environment in NASH and promoting tissue integrity (45). NK cells produce IFN-γ to differentiate Mϕ into M1-like phenotype (non-M2-like phenotype), which plays an anti-fibrogenesis role. Loss of NKp46^+^ cells promotes the development of fibrosis and promotes profibrotic gene expression and a distorted M2 Mϕ phenotype (49).

#### 2.3.2 ILC1

ILC1 was initially identified in the liver of C57BL/6 mice and subsequently in other tissues such as salivary glands, skin, and uterus. Conventional NK cells and ILC1 can be distinguished by their T-box transcription factor properties. The T-box protein in T cells, Tbet, encoded by the Tbx21 gene is involved in IFN-γ production (50, 51). Mature NK cells are strictly dependent on Eomes(Tbet^+^ Eomes^+^) and have a Lin^-^ CD45^+^ NK1.1^+^ NKp46^+^ CD49a^-^ CD49b^+^ phenotype in mice, while ILC1 is independent of Eomes(Tbet^+^ Eomes^-^). The phenotype in mice is Lin^−^ CD45^+^ NK1.1^+^ NKp46^+^ CD49a^+^ CD49b^−^ (52).

Hepatic NKp46^+^ cells account for approximately 10% to 20% of the total number of intrahepatic lymphocytes in mice and 40% to 50% of the total number of intrahepatic lymphocytes in humans (53). Previous studies have shown that high-fat diet (HFD) stimulation can activate adipose tissue and increase the number of ILC1, which is the main source of IFN-γ and TNF-α. At the same time, ILC1 polarizes ATM-M1, leading to adipose tissue inflammation and insulin resistance (54). In addition, ILC1 plays at least in part the role of proinflammatory effector cells in aggravating hepatic ischemia-reperfusion injury(IRI) under steatosis conditions (55).

#### 2.3.3 ILC2

Interleukin-33(IL-33), a cytokine of the IL-1 family, is released extracellularly in chronic hepatocyte stress and marks the accumulation and activation of ILC2 in the liver. Attenuating IL-33-dependent accumulation and activation of ILC2 can inhibit the activation of hepatic stellate cells and reduce the secretion of collagen and matrix proteins, thereby alleviating the degree of liver fibrosis (56). Liver-activated ILC2 produce IL-13, which in turn triggers HSC activation and transdifferentiation in a transcription factor-dependent manner, and participates in the progression of liver fibrosis (57). Meanwhile, Gonzalez-Polo et al. (58) studied liver tissues from patients with liver biopsy and found that ILC2 numbers are significantly increased in fibrotic tissues, activated in tissues that promote human liver fibrosis immunopathology, independent of etiology, and may be a potential new therapeutic target. However, because the interaction between the immune system and lipid metabolism is very complex, it has been shown that lipid metabolism can affect the role of ILC2 in airway inflammation and lung homeostasis. After ILC2 activation, the uptake of fatty acids (FA) is increased, and the utilization of lipid metabolism is significantly increased (59–61).

#### 2.3.4 ILC3

The proportion of ILC3 in the liver is very small. In 2018, Wang et al. (62) showed that ILC3 can promote the formation of liver fibrosis by producing IL-22. However, subsequent studies have focused on attenuating hepatic steatosis by activation of ILC3 to produce IL-22, the major cellular source in the liver, by a high-fat diet; in other words, IL-22 secreted by ILC3 is protective against MASLD spectrum disorders. It has also been confirmed in preclinical models of MASLD that systemic treatment with IL-22 can improve hepatic steatosis, reduce human serum triglyceride levels, reduce intestinal lipid metabolism and uptake, and have liver protective effects (63–66). Hamaguchi et al. (67) found a significant increased M1 macrophages and decreased M2 macrophages in HFD-fed mice. HFD stimulates the production of IL-23 by M1 macrophages, thereby promoting the proliferation of hepatic ILC3, while IL-22 secreted by ILC3 contributes to the up-regulation of hepatic lipid metabolism and has anti-apoptotic activity. Interestingly, ILC3 deficiency leads to significant liver fibrosis even in the absence of HFD. IL-22 produced by ILC3 in the gut plays an important role in maintaining the integrity of the intestinal barrier, altering lipid metabolism and reducing metabolic imbalance (68, 69). High-fat diet can reduce the production of IL-22 by intestinal ILC3, destroy the integrity of the intestinal barrier, and aggravate liver steatosis (70). Although basic research has consistently confirmed that ILC3 proliferation promotes the increase of IL-22 secretion and can reverse MASLD, there is no such study reported in the top Journals. It may be due to the small proportion of ILC3 itself and its short discovery time, so the current research in this field has great potential.

### 2.4 DCs

DCs are the bridge between innate and adaptive immunity and represent the tolerant cell population of the liver in steady state. They are highly heterogeneous, including plasmacytoid dendritic cells, classical type 1 DCs (cDC1; CD103^+^ CD11c^+^ CD11b^-^) and classical type 2 DCs (cDC2; CD103^−^ CD11c^+^ CD11b^+^) (71, 72). A 2022 Mexican study of 128 liver biopsy tissues showed that the frequency of CD11c^+^ DC(cDC) expression in liver tissues of MASLD patients was higher than that of mildly obese or non-obese patients (73). In basic research, Heier et al. demonstrated that cDC1s in mouse liver are a protective cell type that can regulate the influx of inflammatory cells and coordinate the pro-inflammatory and anti-inflammatory environment during the progression of steatohepatitis in mice (74). In parallel, the abundance of cDC1s in visceral adipose tissue is reduced in mice fed a high-fat diet and that reduced expression of cDC1-related genes is characteristic of mice prone to weight gain (75). In addition, deficiency of cDC1s reduced energy expenditure and caused adipose tissue inflammation in middle-aged mice, which was associated with impaired glucose tolerance, insulin resistance, dyslipidemia and hepatic steatosis. The mechanism may be related to the deficiency of and increased phosphorylation of liver kinase B1 (LKB1) in cDCs (76). However, the mechanism of DCs in MASLD spectrum diseases is not fully understood, and more studies are needed to reveal it.

## 3 Emerging therapies targeting innate immune cells in MASLD

MASLD is a systemic disease involving the crosstalk between liver, adipose tissue, muscle and intestinal tract. The key players in the intrahepatic crosstalk are hepatocytes, HSCs, liver sinusoidal endothelial cells and immune cells. At present, the focus of clinical research is mainly on macrophages and NK cells.

Future therapeutic strategies for macrophages may be aimed at changing macrophages from a pro-inflammatory phenotype to a restorative phenotype, but there are no relevant clinical studies. A recent phase 2 open-label randomized controlled trial demonstrated the safety, feasibility, and potential efficacy of using ex vivo-mature autologous monocyte-derived macrophages in patients with cirrhosis (77). In addition, CAR macrophages with antifibrotic T-cell immunity have been developed and shown to be effective in reducing experimental fibrosis (78).

Chimeric antigen receptor (CAR) -modified NK cells will become an effective strategy for the treatment of HCC and hepatic fibrosis in the next decade due to their specificity and low side effects (79). At present, clinical trials have focused on the efficacy of NK cells in the treatment of HCC and hepatitis virus infection(ClinicalTrials.gov, e.g., NCT05040438, NCT04162158, NCT05171309, and NCT03761875, accession date: 28 March 2023).The above studies provide hope for reversing liver fibrosis and supporting liver regeneration.

## 4 Conclusion and perspective

This article reviews the research progress of various innate immune cells in MASLD spectrum diseases, including macrophages, neutrophils, ILCs (ILC1, NK, ILC2, ILC3), and DCs. In the liver, the proportion of different types of innate immune cells ranges from high to low: macrophages/KCs, NK cells, DCs, neutrophils, ILC1, ILC2, and ILC3. Innate immune cells also include mast cells, eosinophils, basophils, etc. No relevant research has been reported when searching the literature, so they are not described in this paper. We used VOSviewer software to analyze all the articles published on the Web of Science from 2000 to February 25, 2025, trying to find the current research hotspots of the role of innate immune cells in MASLD. In this study, VOSviewer software was used to analyze the literature in the Web of Science Core Collection (WoSCC) database from 2000 to February 25, 2025, and try to find the current research hotspots of the role of innate immune cells in MASLD. The search formula used is as follows: (TS =((“Innate immunity cell”) OR (“Congenital immunity cell”) OR (“Nonspecific immunity cell”) OR (“Non-Specific Immunity cell”) OR (“Native Immunity cell”) OR (“Macrophage”) OR (“Dendritic cell”) OR (“Neutrophil”) OR (“Eosinophil”) OR (“Basophil”) OR (“ILCs”) OR (“Innate Lymphoid Cells”) OR (“NK cell”) OR (“ILC1”) OR (“ILC2”) OR (“ILC3”) OR (“Kupffer cell”) OR (“Kupffer cell”)) AND TS=((“non-alcoholic fatty liver disease”) or (“NAFLD”) or (“non-alcoholic fatty liver”) or (“metabolic dysfunction-associated fatty liver disease”) or (“MAFLD”) or (“non-alcoholic steatohepatitis”) or (“NASH”) or (“metabolic dysfunction-associated steatohepatitis”) or (“MASH”) or (“MASLD”))).

**Figure 2:**
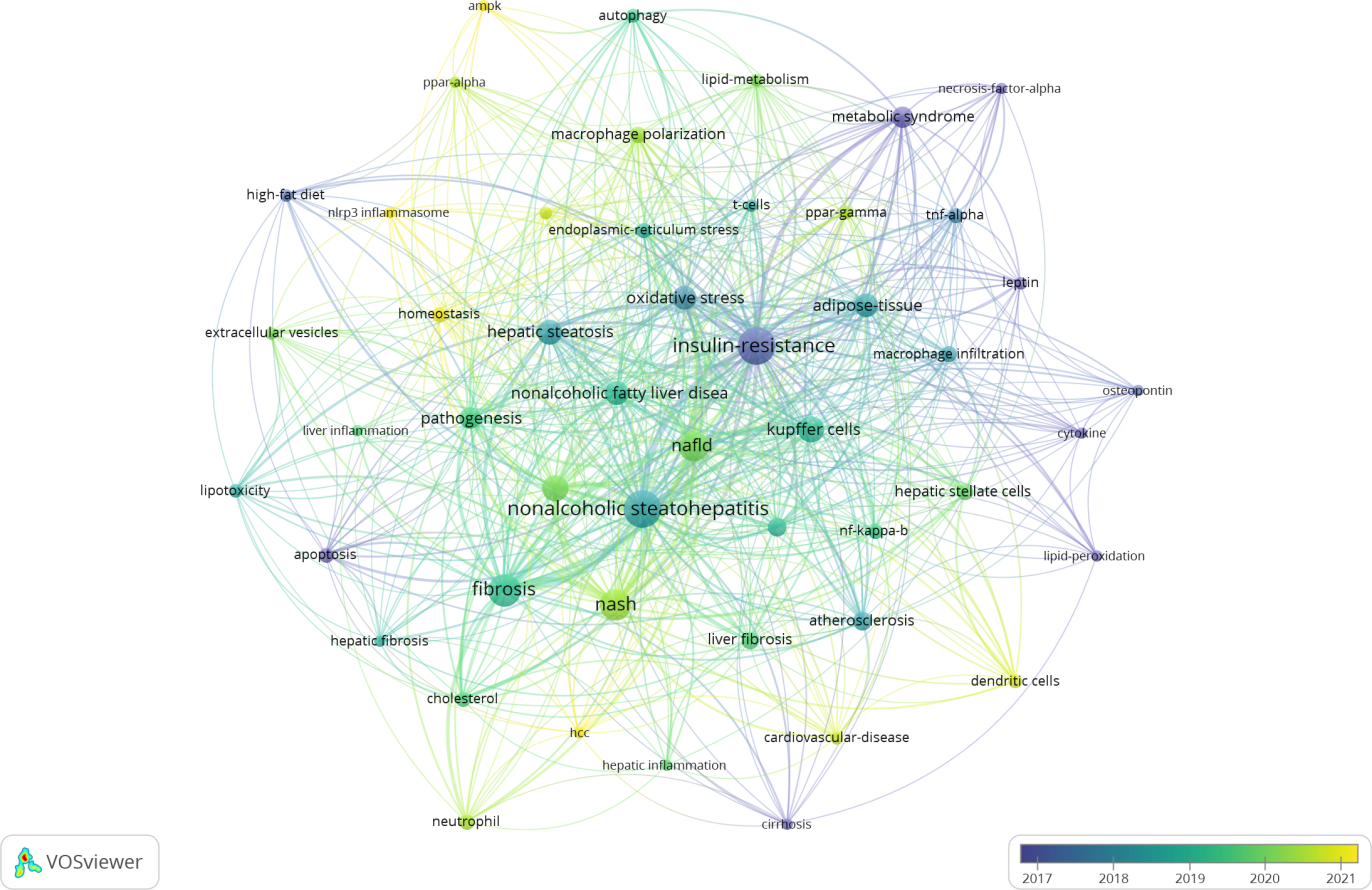
Average citation years for the domains related to innate immune cells and MASLD based on keyword classification.

A total of 1578 articles focused on the relationship between innate immune cells and MASLD. Excluding conference abstracts, editorial materials, book chapters, proceeding papers, letters, and retracted publications and corrections, 1422 articles were finally included for analysis. Keyword burst detection was next performed using Vosviwer to identify emerging trends. **Figure 2** is the graph obtained from VOSviewer analysis based on the average number of occurrences of all keywords in the published papers. In the figure, blue indicates that keywords appear in earlier literature, while yellow is the opposite. Specifically, yellow represents the research hotspots and trends in recent years. **Figure 2** shows that KCs are still being studied by researchers since their discovery, and their recruitment to the liver, macrophage polarization and inflammatory mechanism have been relatively clear (80). Recently, neutrophils, DCs, intestinal microbes, HSCs, peroxisome proliferators-activated receptors-gamma(PPAR-γ), homeostasis have been widely studied. nnate immune cells are the core regulators of MASLD-related inflammation. At present, the mechanism of innate immune cells in the progression of MASLD is relatively unknown, and a large number of studies are still needed to clarify, which provides a reference for researchers in the future research direction.

In addition, it is worth mentioning that the rapid progress of single-cell and spatial omics has enabled us to more precisely distinguish the metabolic characteristics and intercellular crosstalk of various immune cells in the liver of MASLD patients, thus leading to significant changes in our understanding of disease pathogenesis. In particular, the single-cell sequencing of 10 livers (n=5 healthy and n=5 cirrhotic livers) published in 2019 by Ramachandran et al. (81), is known as the benchmark. This article provides an online platform link to quickly identify the changes of each gene in each immune cell, which provides a solid foundation for subsequent research. The development of artificial intelligence such as machine learning makes it possible to use publicly available datasets for cross-merging analysis to find stable potential biomarkers in different cohorts, which is of great significance for early clinical work to identify MASH (82). In the future, we should learn to use omics data to clarify the mechanism of innate immune cells in the progression of MASLD, and work more deeply on the way of preclinical research to clinical transformation. By intervening in the immune mechanism in the process of MASLD, the MASLD patient population and the incidence of MASLD-HCC should be reduced.
